# Clinical Assessment of a Novel Ring Gantry Linear Accelerator-Mounted Helical Fan-Beam kVCT System

**DOI:** 10.1016/j.adro.2021.100862

**Published:** 2021-12-01

**Authors:** Christian Velten, Lee Goddard, Kyoungkeun Jeong, Madhur K. Garg, Wolfgang A. Tomé

**Affiliations:** a Department of Radiation Oncology, Montefiore Medical Center, Bronx, New York; b Institute for Onco-Physics, Albert Einstein College of Medicine, Bronx, New York

## Abstract

**Purpose:**

To assess clinically relevant image quality metrics (IQMs) of helical fan beam kilovoltage (kV) fan beam computed tomography (CT).

**Methods and Materials:**

kVCT IQMs were evaluated on an Accuray Radixact unit equipped with helical fan beam kVCT to assess the capabilities of this newly available modality. kVCT IQMs were evaluated and compared to a kVCT simulator and linear accelerator-based cone beam CTs (CBCT) using a commercial CBCT image quality phantom. kVCTs were acquired on the Accuray Radixact for all combinations of kVp and mAs in fine mode using a 440-mm field of view (FOV). Evaluated IQMs were spatial resolution, overall uniformity, subject contrast, contrast-to-noise ratio (CNR), and effective slice thickness. Imaging dose was assessed for planar kV imaging.

**Results:**

On this kVCT system spatial resolution and contrast were consistent across all settings with 0.28 ± 0.03 lp/mm and 9.8% ± 0.7% (both 95% confidence interval). CNR strongly depended on selected mode (views per rotation) and body size (mA per view) and ranged between 7.9 and 34.9. Overall uniformity was greater than 97% for all settings. Large FOV was not found to substantially affect the IQMs whereas small FOV affected IQMs due to its effect on pitch. Technique-matched CT simulator scans were comparable for uniformity and contrast, while spatial resolution was higher (0.43 ± 0.06 lp/mm), and CNR was between 4% (140 kVp) and 51% (100 kVp) lower. For kV-CBCT, spatial resolutions ranging from 0.37 to 0.44 lp/mm were achieved with comparable contrast, CNR, and uniformity to kVCT. All kVCT scans exhibit imaging artifacts due to helical acquisition. Clinical acquisitions of megavoltage (MV) CT, kV-CBCT, and kVCT on the same patient showed improved and comparable image quality of kVCT compared to MVCT and kV-CBCT, respectively.

**Conclusions:**

Helical fan beam kVCT allows for daily image guidance for localization and setup verification with comparable performance to existing kV-CBCT systems. Scan parameters must be selected carefully to maximize image quality for the desired tasks. Due to the large effective slice thicknesses for all parameter combinations, kVCT scans should not be used for simulation or planning of stereotactic procedures. Finally, improved image quality over MVCT has the potential to greatly improve manual and automated adaptive monitoring and planning.

## Introduction

Image-guided radiation therapy has enabled significant setup margin reduction and has increased the effectiveness of motion management,^1^, ^2^, ^3^ contributing to improved outcomes and reduced toxicities.^4^^,^^5^ Especially powerful are volumetric imaging techniques such as computed tomography (CT) using in-room diagnostic CT scanners, kilovoltage (kV) and megavoltage (MV) cone beam CT (CBCT), and helical fan beam MVCT.^6^, ^7^, ^8^, ^9^

Accuracy and quality of deformable image registration are highly dependent on the quality of the registered studies^10^ and the modalities of the imaging studies, where single-modality registrations tend to be more accurate.^11^ Similarly, intramodality variations in image quality, artifacts, and limitations between kVCT and MVCT or fan beam and CBCT affect deformable image registration quality.^12^, ^13^, ^14^ While these variations can be sufficiently small with no significant loss of quality for kV CT-CBCT registrations,^15^, ^16^, ^17^ significant differences can exist for kVCTMVCT registrations that require manual intervention or postprocessing to enhance registration and contour propagation accuracy.^18^^,^^19^ Thus, volumetric kV imaging is the most desirable technique to increase accuracy and precision for online or offline adaptive radiation therapy workflows that aim to automate image registration, contouring, dose accumulation, and replanning.^20^, ^21^, ^22^ kV-CBCTs can be limited in their field of view (FOV) and suffer from specific cone beam artifacts that can limit their use in areas having multiple air pockets.

Recently, helical fan beam kVCT was made available for Radixact Tomotherapy units under the brand name ClearRT. This modality offers superior image quality over MVCT, due to its lower imaging energy, and over CBCT, due to its fan-beam like geometry. Additionally, scan lengths up to 135 cm are available and scan times are greatly reduced when compared to MVCT acquisitions. One of these systems has been installed and commissioned for clinical use on our department's Accuray Radixact system.

Planar kV imaging was first made available on the Radixact with the Synchrony system (Gen1), which allows for motion compensation during treatment delivery. For users who were previously using Synchrony on Radixact, the ClearRT upgrade requires changes to the x-ray imaging hardware, possibly affecting image quality and dose. This study aims to compare the performance of this second generation (Gen2) x-ray imaging system for planar and volumetric imaging with the Gen1 system and to assess its clinical performance by comparing helical fan beam kVCT with a technique-matched CT-simulator, CT-simulator kVCT using standard simulation protocols, and 2 other linear accelerator-based CBCT systems.

## Methods and Materials

To assess the clinical effect of the helical fan beam kVCT system, relevant image quality metrics (IQM) were compared between the Accuray Radixact kVCT, Accuray Radixact MVCT, Varian TrueBeam kV-CBCTs, Varian Halcyon kV-iCBCT, and a diagnostic grade 4-slice GE LightSpeed RT CT simulator (CT-Sim). It should be noted that some of the usage modes tested in this work are only available with a ClearRT kV max license and as such may not be available to all users.

### Description of the ClearRT system

The ClearRT system provides an alternative imaging modality for patient setup. It allows the user to select imaging parameters by anatomical site, body size, required FOV, and mode. The options and their effects on the imaging procedure are described below.

Like MVCT, the user can choose between fine, normal, and coarse modes, but unlike MVCT this does not predictably affect pitch or slice thickness. With ClearRT choice of mode affects the beam width at isocenter, couch travel speed, and the number of views (x-ray projections) per rotation, which are 600, 480, and 360 for fine, normal, and coarse mode, respectively. Pitch is only affected by mode choice when using the small FOV (270 mm), at 0.86 for fine, 1.0 for normal, and 1.4 for coarse, while for an FOV of 440 mm or 500 mm, pitch is fixed at 0.75 for all modes. Imaging speed increases progressively from fine to coarse mode due to the larger beam width at isocenter that necessitates a higher couch travel speed. With a 440 mm FOV imaging speeds are 27.4 cm/min, 54.7 cm/min, and 84.4 cm/min for fine, normal, and coarse modes, respectively. For instance, a 30-cm long scan would take 65.7 s in fine, 32.9 s in normal, and 21.3 s in coarse mode. Fewer views per rotation and increased beam width at the imaging isocenter increase the scatter contribution and CT cone angles. This introduces imaging artifacts and leads to a decrease in image quality.

The choice of anatomical site determines the peak tube potential where head, thorax, and pelvis correspond to 100 kVp, 120 kVp, and 140 kVp, respectively. Similarly, the choice of body size determines the tube current per view where small, medium, large, and x-large (not available with 100 kVp) correspond to 80 mA, 125 mA, 160 mA, and 200 mA, respectively. The system reports the total mAs per rotation, which is affected by mA (body size) and number of views per rotation (mode) with a constant exposure time per view of 5 ms/view. Depending on the FOV the beam is filtered using a half bowtie filter (440 and 500 mm) or 0.5 mm copper filter (270 mm).

### Gen2 kV system performance

Helical fan beam kVCT, which has become available on the Gen2 system, utilizes updated x-ray imaging hardware that replaces the Gen1 kV imaging system used for planar kV imaging with the Synchrony system on the Radixact.

To allow for helical fan beam kVCTs to be performed with a range of beam widths, collimators were added to the kV source. The ClearRT imaging detector panel is of higher resolution compared to the Gen1 Synchrony panel. The source and detector positioning have been changed to allow for the Gen2 hardware to be fitted onto the ring gantry; in the Gen2 system the updated imager hardware allows for the imager panel to be positioned off-center to accommodate the larger 440 mm and 500 mm FOVs. Briefly, the ClearRT imaging system has a 150 µm pixel size, binned to 450 µm for planar imaging. The nominal source to image distance is 155 cm and the magnification factor is 1.49 at isocenter.

X-ray parameters were measured for thorax and pelvis protocols of all body sizes (XXS through XXL) using a PTW NOMEX multimeter (PTW, Freiburg, Germany) positioned at isocenter on 10 cm of solid water with 3 mm of lead shielding its electronics. Measured parameters were mean tube potential (kVp), dose (air kerma) per exposure, half value layer (HVL), and total filtration.

Planar image quality for the Gen1 and Gen2 kV hardware was assessed using a commercial machine quality assurance (QA) phantom, the SNC kV-QA phantom (Sun Nuclear, Melbourne, FL), positioned at isocenter. Relevant IQMs for comparison were spatial resolution, minimum uniformity, contrast, and contrast-to-noise ratio (CNR).^8^^,^^23^

### CT image quality

Image quality was assessed using a commercial CT/CBCT image quality phantom, CatPhan 504 (The Phantom Laboratory, Greenwich, NY), containing different modules for geometry/sensitometry, high resolution, low contrast, and uniformity testing.

Helical fan beam kVCT scans were acquired for all modes, anatomical sites, and body sizes using a 440 mm FOV (Table E1). Additional scans were taken employing the 270 mm FOV for all head sizes using fine mode and using the 500 mm FOV for medium pelvis using fine mode. Finally, fine mode scans of large head and pelvis using small and large FOV, respectively, were acquired in machine QA mode as these settings are not available for selection in clinical mode. MVCT scans were acquired at normal pitch with 2-mm slice thickness for standard and iterative reconstruction techniques to compare the current system's performance to a previous study.^14^

CT simulator scans were acquired for all preset anatomical site techniques, including brain, head and neck, chest/thorax, abdomen, pelvis, and (general) stereotactic body radiation therapy (SBRT) on a GE LightSpeed RT (Table E2). To directly compare the Radixact ClearRT system's performance, additional scans were acquired with techniques matched to the ClearRT parameters (mode: fine, FOV: 440 mm) as closely as possible, including slice thickness, pitch, kVp, and mA (Table E1). Scans were reconstructed using the *standard* convolution kernel. For head and neck, select scans were also reconstructed using *soft, detail*, and *boneplus* kernels.

Kilvoltage-CBCT and iCBCT scans were acquired employing all clinically used modes for head, thorax, and pelvis on a Varian TrueBeam (v2.7) and Varian Halcyon (v2.0) (Table E3). For Halcyon iCBCT, all scans are half-fan due to the fixed x-ray imager geometry.

Several IQMs were evaluated using DoseLab v7.0 (Varian Medical Systems, Palo Alto, CA), including automatically and manually evaluated spatial resolution, contrast, and CNR.^8^^,^^23^ Here, automatic evaluation corresponds to DoseLab's calculation of the modulation transfer function (MTF) and reporting of the number of line pairs per millimeter at which the MTF=0.5. Modulation for each region-of-interest (ROI) (Equation 1) is calculated from the 90^th^ and 10^th^ percentile signal levels and subsequently normalized to the maximum modulation:

(1) $$Modulation=\frac{S_{90}-S_{10}}{S_{90}+S_{10}}$$

Manual evaluation is based on the largest frequency line pairs visible at best contrast as judged by 1 of the authors. Contrast between 2 ROIs (Equation 2) is calculated as

(2) $$C_{2,1}=\frac{\left|S_{2}-S_{1}\right|}{S_{2}+S_{1}}$$

where $S_{1}$ and $S_{2\;}$are the mean pixel values of ROI_1_ and ROI_2_. CNR (Equation 3) is the calculated as contrast divided by combined relative noise in the ROIs:

(3) $$\text{CNR}=C_{2,1}\;/\;{\left(\frac{\sigma_{1}^{2}+\sigma_{2}^{2}}{S_{1}^{2}+S_{2}^{2}}\right)}^{1/2}$$

Finally, slice thickness was measured using the wire ramps in the CTP401 module.

Hounsfield unit (HU) measurements were performed using the Virtual Water Tomophantom (Med-Cal Inc, Verona, WI), which allows for placement of interchangeable density plugs in central and distal rings. Density inserts ranging from 0.28 g/cm^3^ (lung) to 1.821 g/cm^3^ (cortical bone) were utilized for comparison. Machine HU calibrations were performed during commissioning according to the vendor's specifications. Scans were acquired with the phantom centered at isocenter using pelvis protocols (140 kVp) on all 3 machines. Helical fan beam kVCTs were acquired in fine mode with 440 mm FOV and pelvis large setting. kV-(i)CBCTs were acquired using the clinical standard pelvis protocols. Evaluation for each density insert was performed in cylindrical ROIs having 1.5-cm diameter and an approximate length of 1 cm, centered in each density plug. These ROIs were created on 1 scan and subsequently transferred to other scans. Weighted averages were calculated from all measurements of a density insert and minimum, median, and maximum differences from this weighted average reported.

## Results

Operating characteristics of the Gen1 and Gen2 kV imaging system were comparable in terms of mean kVp and HVL, differing by no more than 0.5%, and total filtration, differing on average by less than 2.5% (Table 1). Air kerma per exposure was reduced by over 64% for all protocols for the Gen2 system. Image quality metrics, except for CNR, were consistent between all protocols for each imaging system separately. Contrast was aberrantly high for pelvis (M) for the Gen1 system. For the Gen2 system, spatial resolution decreased, contrast and CNR increased, and minimum uniformity remained consistent within 0.5%. Spatial resolution decreased from an average of 1.14 to 0.97 lp/mm, contrast increased from an average of 40% to 47%, and CNR increased on average by 9.4%. IQMs could not be evaluated using the SNC kV-QA phantom for tube voltages of 140 kV for both the Gen1 and Gen2 systems, and for the Gen2 system at 120 kV and 1.6 mA, as the phantom was not visible using this combination of tube voltage and current.

**Table 1:** kV system operating characteristics before and after the hard- and software upgrade to ClearRT

Protocol	Voltage (kV)	Tube current (mA)	Air kerma per exposure (mGy)	MeankVp	HVL (mmAl)	Total filtration (mmAl)	Spatial resolution (lp/mm)	Minimum uniformity	Contrast	CNR
Before upgrade										
Thorax (XXS)	100	0.8	0.051	102.3	8.9	21.4	1.17	98.3%	38.2%	44.7
Thorax (XS)	100	1.0	0.063	102.4	8.9	21.6	1.17	98.5%	38.1%	49.1
Thorax (S)	120	0.8	0.090	118.0	9.9	21.8	1.17	98.7%	36.2%	51.2
Thorax (M)	120	1.0	0.112	124.3	9.8	19.7	1.17	98.7%	36.5%	53.4
Thorax (L)	120	1.6	0.114	122.3	9.9	20.6	1.16	99.0%	36.9%	64.1
Pelvis (S)	120	1.0	0.140	121.7	9.8	19.8	1.17	98.6%	36.8%	51.4
Pelvis (M)	120	1.25	0.179	123.6	9.9	20.3	0.94	96.9%	60.7%	62.4
Pelvis (L)	120	2.0	0.222	120.2	9.8	20.6	1.17	99.0%	37.7%	64.8
Pelvis (XL)	140	4.0	0.705	142.1	10.9	21.0	-	-	-	-
After upgrade										
Thorax (XXS)	100	0.8	0.018	103.7	8.9	21.4	0.98	97.5%	47.9%	48.5
Thorax (XS)	100	1.0	0.022	103.4	8.9	21.4	0.97	97.7%	48.4%	54.8
Thorax (S)	120	0.8	0.032	119.3	9.9	21.6	0.97	97.9%	47.1%	53.3
Thorax (M)	120	1.0	0.038	124.1	9.9	21.7	0.97	98.0%	48.0%	60.8
Thorax (L)	120	1.6	0.039	122.4	9.8	19.8	-	-	-	-
Pelvis (S)	120	1.0	0.047	120.0	9.9	21.7	0.97	98.1%	47.1%	57.4
Pelvis (M)	120	1.25	0.063	123.5	10.0	21.9	0.97	98.3%	47.9%	66.4
Pelvis (L)	120	2.0	0.075	122.6	9.8	19.5	-	-	-	-
Pelvis (XL)	140	2.5	0.151	144.6	10.87	20.9	-	-	-	-
Pelvis (XXL)	140	4.0	0.238	142.0	10.9	21.4	-	-	-	-

Abbreviations: CNR = contrast-to-noise ratio; HVL = half value layer; kV = kilovoltage.

Entries with “-” could not be evaluated.

Table 2 shows the summary of IQMs for helical fan beam kVCT using fine and coarse modes, as well as the matched CT simulator protocols. Results of CBCT acquisitions for head, thorax, regular and large pelvis along with a comparison to helical fan beam kVCT techniques that have similar closest imaging dose (CTDI_vol_) (Tables E1 and E3) are shown in Table 3.

**Table 2:** Image quality metrics for ClearRT (fine and coarse modes) and technique-matched CT simulator.

		ClearRT kVCT (fine, 440 mm FOV)										
		Head (100 kV)			Thorax (120 kV)				Pelvis (140 kV)			
		Small	Medium	Large	Small	Medium	Large	X-large	Small	Medium	Large	X-large
Spatial resolution	(lp/mm)	0.28	0.28	0.28	0.28	0.28	0.30	0.28	0.27	0.29	0.28	0.28
Manual spat. res.	(lp/mm)	0.45	0.45	0.45	0.45	0.45	0.45	0.45	0.40	0.40	0.40	0.40
Overall uniformity	(%)	98.8	99.0	99.2	99.0	99.0	99.3	99.1	99.0	99.1	99.0	99.2
Contrast	(%)	10.1	10.3	10.2	10.2	9.9	9.9	10.0	9.8	9.9	10.0	9.9
CNR		18.2	23.7	29.1	24.8	30.7	32.7	30.4	22.8	29.9	31.7	34.9
Slice thickness	(mm)	4.0	3.9	3.9	4.5	4.6	4.5	4.8	4.2	4.5	4.4	4.7
		ClearRT kVCT (coarse, 440 mm FOV)										
		Head (100 kV)			Thorax (120 kV)				Pelvis (140 kV)			
		Small	Medium	Large	Small	Medium	Large	X-large	Small	Medium	Large	X-large

Spatial resolution	(lp/mm)	0.26	0.28	0.27	0.26	0.27	0.27	0.26	0.27	0.26	0.25	0.26
Manual spat. res.	(lp/mm)	0.40	0.40	0.40	0.40	0.40	0.40	0.40	0.40	0.40	0.40	0.40
Overall uniformity	(%)	97.6	97.3	97.9	97.3	97.8	97.8	97.4	97.2	98.0	98.0	98.2
Contrast	(%)	9.4	9.5	9.7	9.4	9.7	9.3	9.6	9.7	9.6	9.6	9.5
CNR		7.9	11.3	13.1	11.8	11.0	14.7	11.4	11.9	12.7	14.6	14.3
Slice thickness	(mm)	4.1	3.7	4.3	3.9	5.4	4.5	4.4	5.8	5.2	5.0	4.6
		Matched CT-simulator										
		Head (100 kV)			Thorax (120 kV)				Pelvis (140 kV)			
		Small	Medium	Large	Small	Medium	Large	X-large	Small	Medium	Large	X-large

Spatial resolution	(lp/mm)	0.45	0.45	0.40	0.39	0.43	0.46	0.41	0.39	0.42	0.42	0.40
Manual spat. res.	(lp/mm)	0.65	0.65	0.65	0.60	0.65	0.65	0.60	0.60	0.65	0.60	0.60
Overall uniformity	(%)	98.3	98.5	98.8	99.2	99.4	99.4	99.5	99.3	99.5	99.6	99.6
Contrast	(%)	10.3	10.4	10.5	10.2	10.2	10.2	10.2	10.0	10.0	10.0	10.0
CNR		10.1	12.6	14.4	20.4	23.3	24.0	24.8	19.7	21.8	30.5	31.8
Slice thickness	(mm)	2.3	2.7	2.7	4.0	4.0	4.0	3.6	3.9	3.8	4.1	4.0

Abbreviations: CNR = contrast-to-noise ratio; CT = computed tomography; FOV = field of view; kV = kilovoltage.

**Table 3:** Image quality metrics for clinically used patient setup imaging

		Head			Thorax			Pelvis			Pelvis large		
		ClearRT (coarse-S)	CBCT	iCBCT	ClearRT (coarse-S)	CBCT	iCBCT	ClearRT (normal-L)	CBCT	iCBCT	ClearRT (fine-XL)	CBCT	iCBCT
Spatial resolution	(lp/mm)	0.26	0.43	0.45	0.26	0.38	0.37	0.27	0.37	0.37	0.28	0.37	0.36
Manual spat. res.	(lp/mm)	0.40	0.70	0.60	0.40	0.50	0.45	0.45	0.70	0.40	0.40	0.50	0.40
Overall uniformity	(%)	97.6	95.2	96.5	97.3	98.0	97.6	98.7	94.6	99.0	99.2	98.5	99.0
Contrast	(%)	9.4	10.7	10.3	9.4	10.5	10.5	9.7	10.3	10.5	9.9	10.4	10.4
CNR		7.9	4.1	4.4	11.8	11.2	7.90	18.4	14.2	53.8	34.9	20.4	61.7
Slice thickness	(mm)	4.1	2.4	2.3	3.9	2.6	2.4	4.3	2.5	3.0	4.7	2.5	2.5

Abbreviations: CBCT = cone beam computed tomography; CNR = contrast-to-noise ratio.

ClearRT modes were chosen based on the closest imaging dose (CTDI_vol_).

HU mean and standard deviation for each ROI are tabulated in Table E4. HU standard deviations ranged from 9 to 17 HU on fine mode fan beam kVCT scans, from 14 to 34 HU on iCBCT scans, and from 16 to 44 HU on CBCT scans. Weighted average HUs were 484 ± 8, 852 ± 9, and 1294 ± 14 for 30%, 50%, and 100% cortical bone, respectively. The Lung LN-300 and LN-400 inserts had average HUs of –707 ± 12 and –527 ± 8. Water-equivalent plastic inserts had average HUs of 18 ± 8 and 31 ± 7 at central and distal positions, respectively, while true water inserts had average HUs of 30 ± 8 and –11 ± 7 at these distances. Differences from these weighted averages ranged from –10 to 21 (average 8) for helical fan beam kVCT, from –131 to 5 (average –52) for iCBCT, and from –45 to 31 (average 36) for CBCT. Differences exceeding 50 HU were found for 30% to 100% cortical bone on iCBCT with ΔHU<−122 and CBCT with ΔHU>51, as well as for true water at larger distance from the rotation axis on iCBCT with ΔHU=−58.

**Fig. 1:**
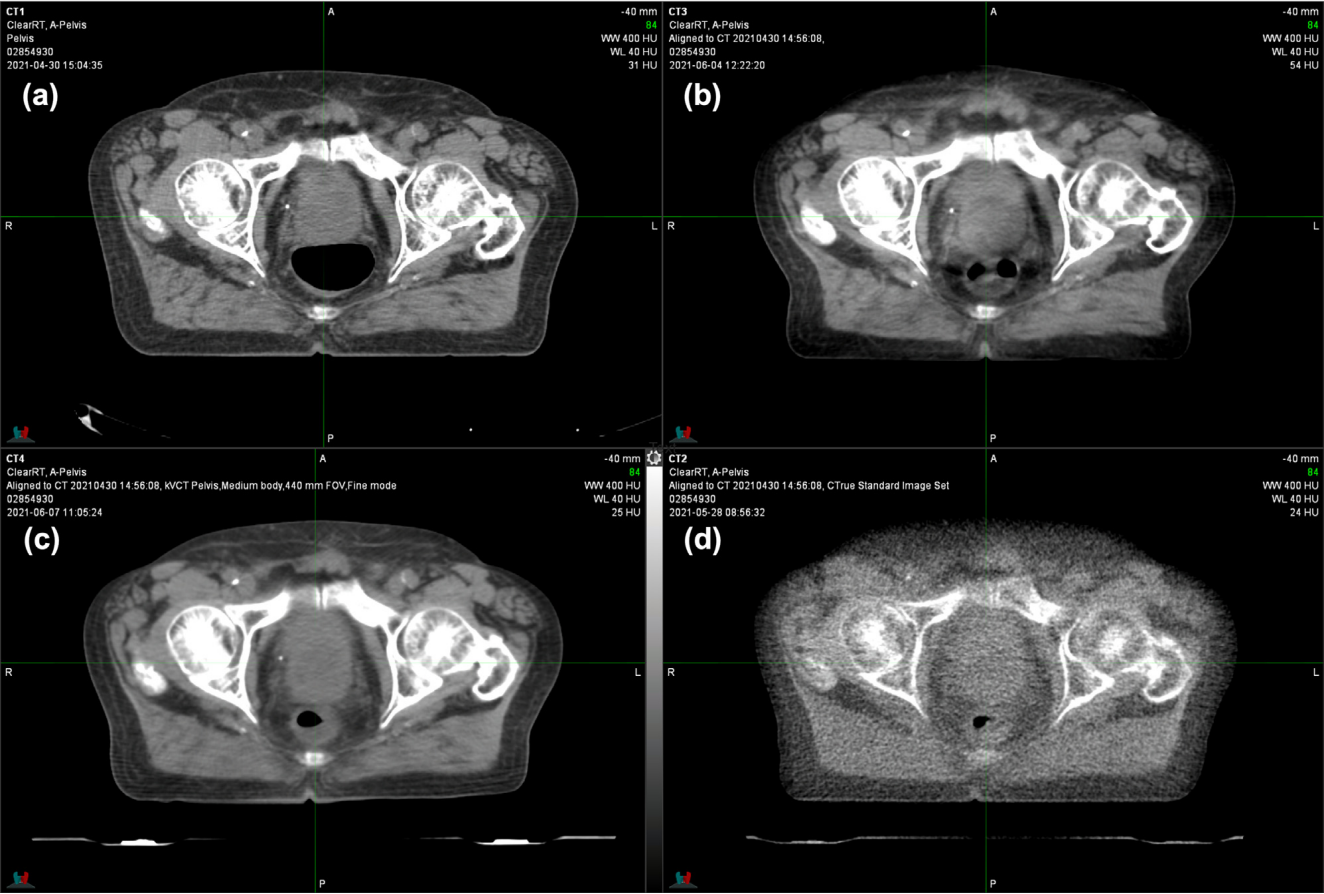
Computed tomography (CT) simulation **(a)**, kilovoltage cone beam CT (kV-CBCT) **(b)**, ClearRT kVCT **(c)**, and megavoltage (MV) CT **(d)** of a patient treated for prostate cancer. The ClearRT scan was acquired with pelvis medium settings in fine mode showing comparable contrast and spatial resolution to CBCT.

**Fig. 2:**
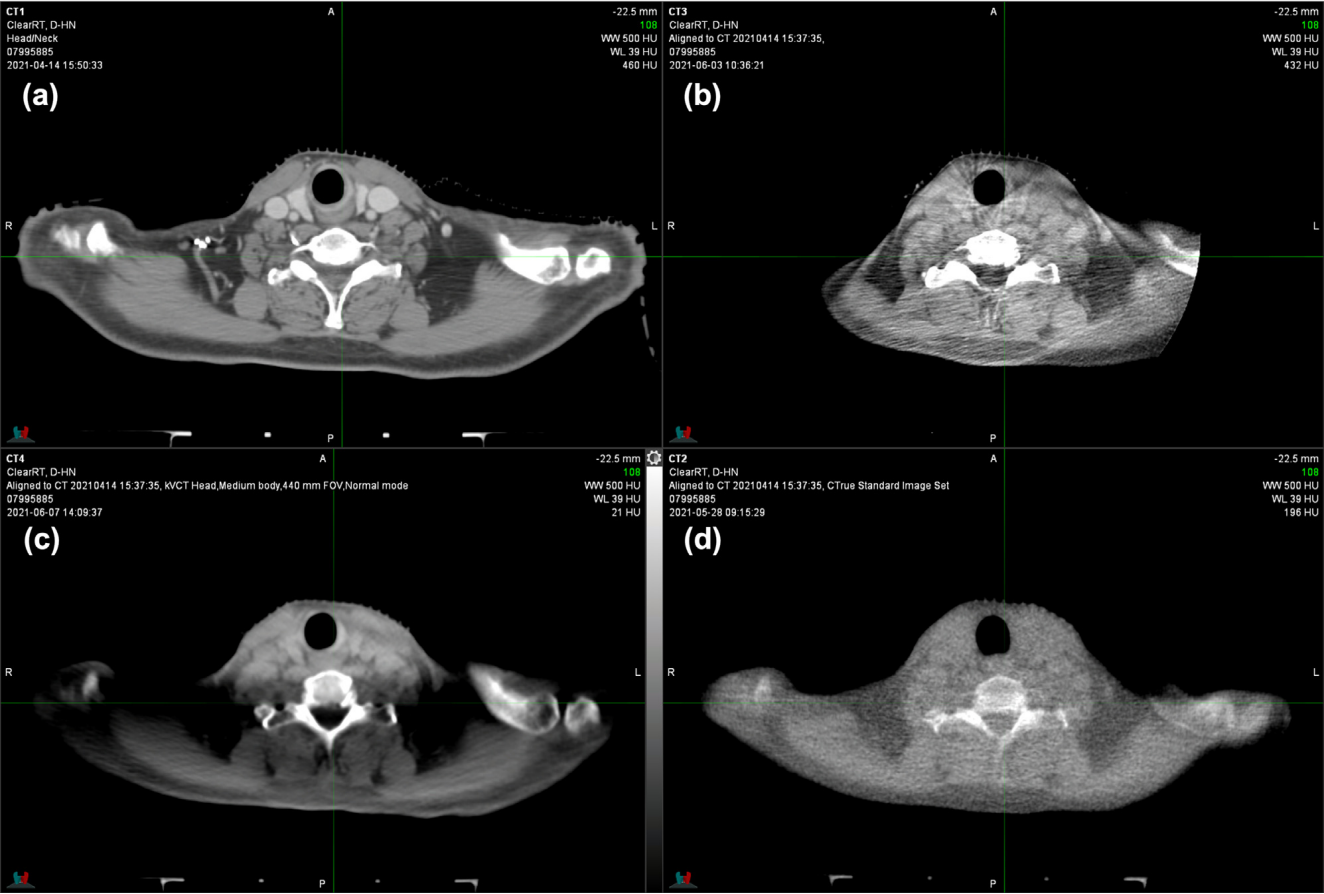
Computed tomography (CT) simulation **(a)**, kilovoltage cone beam CT (kV-CBCT) **(b)**, ClearRT kVCT **(c)**, and megavoltage (MV) CT **(d)** of a patient treated for head and neck cancer of the larynx. The ClearRT scan was acquired with head medium settings in normal mode showing considerable loss of signal between the highly attenuating bony anatomy. The CBCT was acquired in full-fan head mode with limited field of view, exhibiting minor CBCT and photon starvation artifacts.

**Fig. 3:**
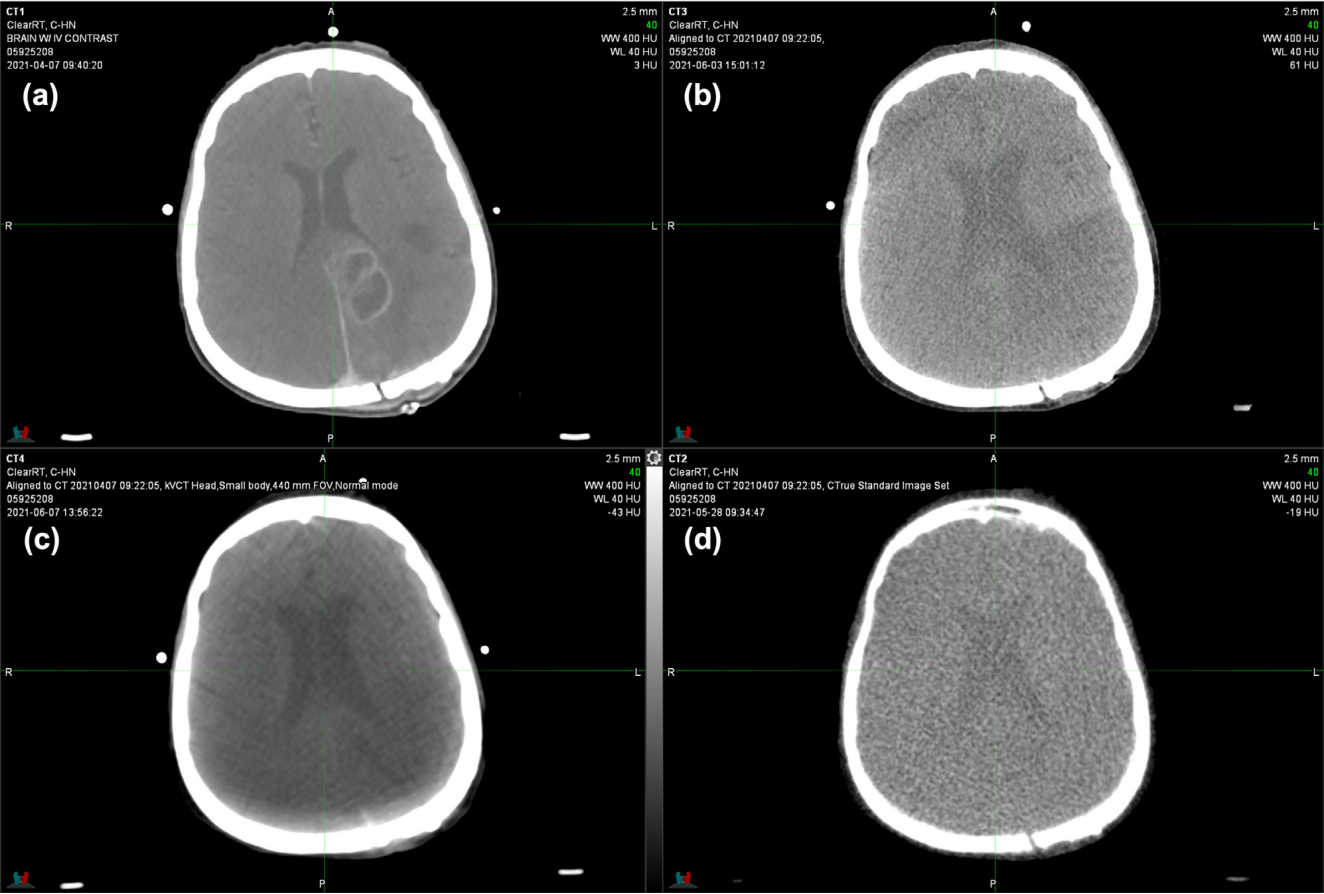
Computed tomography (CT) simulation **(a)**, kilovoltage cone beam CT (kV-CBCT) **(b)**, ClearRT kVCT **(c)**, and megavoltage (MV) CT **(d)** of a patient treated for grade 4 glioma. The ClearRT scan was acquired with head small settings in normal mode exhibiting windmill and beam hardening artifacts in brain tissue. Notably, the craniotomy defect in the left posterior cranium is not visible only on the ClearRT scan. The CBCT shows qualitatively similar noise levels in soft tissue to those of MVCT.

Several patients were treated over the course of the upgrade on a C-arm linear accelerator (Varian TrueBeam). For these patients, treatment planning CT and MVCT and CBCT and kVCT daily image guidance scans are available for comparison. CT simulator (a), kV-CBCT (b), helical kVCT (c), and helical MVCT (d) scans are shown in Figures 1, 2, and 3 for patients undergoing treatment for prostate, head and neck, and brain cancer, respectively.

## Discussion

Comparisons of kV system parameters have shown that the Gen2 system has similar operating characteristics (kVp, HVL, filtration) as the Gen1 system that was used for Synchrony on Radixact. Large reductions in air kerma per exposure were found for the Gen2 kV system. This is due to the addition of a 0.5-mm copper filter, increased source-to-axis distance from 57.5 to 104 cm, and decreased axis-to-detector distance from 56 to 51 cm. These changes in source/detector geometry could lead to a 42% reduction in dose to achieve the same signal level at the detector, based on the inverse square law, with additional dose reductions due to the removal of low energy photons with the copper filter. These air kerma values are consistent with those reported in the vendor's reference material. Similarly, the degraded spatial resolution from an average of 1.14 to 0.97 lp/mm can be explained by the change in magnification, the ratio of source-to-detector distance and source-to-axis distance, which changed from 1.97 to 1.49, and the projected effective pixel size at isocenter, which increased from 0.20 µm to 0.30 µm. Despite the increased resolution of the Gen2 detector panel, pixel binning is performed for planar imaging with the Gen2 system, affecting spatial resolution. While the spatial resolution of the Gen2 kV system is reduced it is important to note that the primary use of planar kV imaging is for intrafraction motion tracking using Synchrony, where improvements in contrast and CNR along with a reduced imaging dose are more important to reliably identify and track either fiducials or the tumor itself, as in the case of fiducial free tracking of lung lesions.^24^^,^^25^

Imaging dose, as reported using CTDI_vol_ for helical fan beam kVCT fine mode scans, compared favorably to that of technique-matched CT simulator scans. For head, thorax, and pelvis protocols, dose indices were 10%, 21%, and 18% to 23% lower, respectively.

Contrast was consistent between all evaluated helical fan beam kVCT modes with an average of 9.8% (±0.7%, 95% confidence interval [CI]), all CT simulator scans with an average of 10.2% (±0.5%, 95% CI), and regular and iterative CBCT with an average of 10.5% (±0.4%, 95% CI). Contrast is measured between the proprietary Delrin and water-equivalent material within the Catphan 504 phantom, which have effective atomic numbers of 6.95 and 7.42 according to the vendor's reference. Thus, changes in the beam energy spectrum with tube voltage are expected to have minimal impact on the attenuation characteristics of these materials.

For all modalities, CNR increased with increasing mA and number of views per rotation, or more simply, with mAs. This is expected, as an increased photon fluence reduces statistical noise, increasing CNR at consistent contrast. For helical fan beam kVCT the smallest and largest CNRs for fine mode were 18.2 (head, small) and 34.9 (pelvis, x-large); with coarse mode, the smallest and largest CNRs were 7.9 (head, small) and 14.6 (pelvis, large). For matched CT-simulator techniques the extreme CNR values were 10.1 (head, small) and 31.8 (pelvis, x-large), while for clinically used protocols CNR was in the range of 10.0 to 15.5 for thoracic, abdominal, and pelvic protocols and between 19.2 and 23.2 for brain, head and neck, and SBRT protocols. CNR values for CBCT and iCBCT were lowest for head protocols, with 4.1 and 4.4, and highest for large pelvis protocols, with 20.4 and 61.7, respectively (Table 3). CNR almost doubled (80%-102%) for head scans at 100 kVp compared to the CT simulator. For thorax and pelvis scans the increase in CNR was more moderate, 22% to 36% and 4% to 37%, respectively.

Spatial resolution was found to be consistent for helical fan beam kVCT modes with 0.28 lp/mm (±0.03, 95% CI) and matched CT simulator scans with 0.43 lp/mm (±0.06, 95% CI). For conventional clinical CT simulator protocols spatial resolution was slightly lower at 0.38 lp/mm (±0.04, 95% CI), while for the SBRT protocol it was 0.45 lp/mm. This is expected, as the SBRT protocol performs the scan in axial mode, avoiding any reduction in spatial resolution due to helical scanning. Spatial resolution was consistent between CBCT and iCBCT for the same protocols with 0.43 to 0.45 lp/mm for head and 0.36 to 0.38 lp/mm for thorax, pelvis, and large pelvis. These trends also occur when comparing manually evaluated spatial resolution. Compared to other modalities, spatial resolution for helical fan beam kVCT was inferior for all modes, with 25% to 38% fewer lp/mm for both automatically and manually evaluated spatial resolution. Together with the consistent contrast between helical fan beam kVCT and CT simulator, this suggests that the reconstruction algorithm used in the helical fan beam kVCT system employs noise reduction techniques that in turn reduce spatial resolution. This behavior was observed previously when comparing MVCT image quality between different reconstruction modes.^14^

These differences in CNR and spatial resolution carried over to the comparison with clinically used simulation protocols for thoracic, abdominal, and pelvic sites. For brain and head and neck protocols CT simulator CNR was comparable to helical fan beam kVCT small/medium head scans in fine mode, with CNRs of 19.2 to 23.2 compared to 18.2 to 23.7.

The use of the coarse mode was found to yield IQMs similar to those previously found for MVCT,^14^ with CNR reduced by 50% or more, slightly decreased uniformity and contrast, and increased effective slice thickness compared to fine mode.

Furthermore, scans on larger uniform cylindrical phantoms showed differences in average CT number between the beginning and center of the scan, in the axial direction, of approximately 175 HU. This is likely due to the larger cone angles used in the reconstruction for coarse mode and the large effective slice thickness on the helical fan beam kVCT system. This effect would require an increased scan length, effectively increasing imaging dose and scan time, negating reductions in imaging dose from fewer views per rotation and less scan time from a larger longitudinal FOV with coarse mode. By comparison, normal mode may strike a good balance between IQMs, imaging dose, and scan time, and possibly yield best results for daily image guidance.

Slice thicknesses measured with the wire ramp were larger than the nominal slice thickness for all modalities. Measured versus nominal slice thicknesses were 4.0 versus 2.4 mm and 4.6 versus 3.6 mm for head and other protocols on helical fan beam kVCT, while for matched CT simulator scans slice thicknesses were 2.5 versus 2.5 mm and 3.9 versus 3.75 mm for head and other protocols. For CBCT, slice thickness was on average 2.5 versus 2.0 mm. These differences in nominal and measured slice thicknesses for all helical and cone beam acquisitions can be attributed to the width, usually full width at half maximum, of the slice sensitivity profiles, which are usually larger than the nominal slice thickness.^26^ The measured thicknesses of 4.0 and 4.6 mm are considerably closer to the sensitivity profile full width at half maximum of 3.6 and 4.2 mm, respectively. Furthermore, it is important to note that while the nominal slice thicknesses are 2.4 and 3.6 mm for head and thorax/pelvis sites, the actual slice spacing is half of that, meaning that the data acquisition volumes of 2 adjacent slices overlap each other by 50%, leading to considerable volume averaging. Thus, these scans should not be used for planning of stereotactic treatments despite their nominal slice spacing of <3 mm due to the reduced accuracy in identifying and delineating small structures.^27^^,^^28^ Similarly, CT simulation protocols using helical acquisition also showed an increased measured slice thicknesses whereas axial acquisition used in the SBRT protocol did not deviate from the nominal slice thickness. kV-CBCT also showed approximately 25% larger effective slice thickness compared to nominal slice thicknesses. Thus, except for axial CT, all modalities suffer from similar relative axial resolution degradation. However, because the standard reconstruction slice thicknesses on our CT simulator and kV-CBCT systems are 2.5 and 2 mm, respectively, the absolute increase in slice thickness and thus volume averaging is more limited for these systems compared to the helical fan beam kVCT.

Image quality metrics of helical fan beam kVCT and kV-CBCT systems for target localization and setup verification were compared to techniques with similar CTDI_vol_ dose indices (Tables E1 and E3). Spatial resolution on kV-CBCT was superior to helical fan beam kVCT for all protocols at 0.44 versus 0.26 lp/mm for head and 0.37 versus 0.26 lp/mm for thorax, pelvis, and pelvis large. CNR was consistent between helical fan beam kVCT, CBCT, and iCBCT for head and thorax protocols. While this metric is almost twice as high for head helical fan beam kVCT scans it should be noted that the CTDI_vol_ of the selected protocol is still double that of the CBCT. For pelvis and pelvis large protocols the iCBCT algorithm improves CNR compared to regular CBCT by a factor of 3 to 3.8, with helical fan beam kVCT showing slightly better results than regular CBCT. Notably, the iCBCT algorithm does so without an observable loss of spatial resolution at the 50% MTF level. Similarly, manual evaluation, which is more closely related to the 10% MTF level, does show some reduction in spatial resolution, albeit not below that of helical fan beam kVCT.

HU variations were smallest for helical fan beam kVCT, with at most 21 HU deviation from the weighted average of all 3 modalities. This result is expected because the investigated mode (fine, pelvis large, 440 mm FOV) was previously calibrated using the same phantom, whereas Halcyon iCBCT and TrueBeam CBCT were calibrated using the 2 vendor-supplied phantoms, namely the QUART phantom (QUART GmbH, Zorneding, Germany) and the CatPhan 504, respectively. This may also explain larger deviations observed for cortical bone, which are largest for Halcyon iCBCT, as the QUART phantom provides only 3 calibration points: air, polystyrene, and CaF_2_. In contrast, the CatPhan 504 provides 2 additional density inserts between polystyrene and CaF_2_: acrylic and Delrin, whose electron densities are closer to those of 30% and 50% cortical bone. Finally, the difference in true water HU at the periphery of the phantom was –58 for iCBCT, which may be due to reconstruction artifacts of the semispherical end of the insert. kVCT scans excluded the superior and inferior 4-cm portions of the phantom, to prevent known artifacts caused by partial irradiation of the phantom/air interface. Artifacts were also seen at the superior and inferior phantom borders of the phantom for the Halcyon iCBCT, which were confirmed by the vendor to be attributable to the reconstruction algorithm introduced due to penumbra from the kV source blades.

Helical fan beam kVCT used for clinical cases provides visibly improved images compared to MVCT with considerably less noise and improved soft tissue contrast (Fig. 1-3). As in the phantom tests spatial resolution is reduced, which shows especially for structures with similar CT numbers (Fig. 1 and 2). The helical fan beam kVCT acquisition of the lower neck region in Figure 2c exhibits extensive reductions in CT numbers in the areas between the cervical spine and humeral heads, which is likely due to increased attenuation and beam hardening for lower kVp protocols. kV-CBCT using the same tube potential is also affected, albeit not as severely, by increased attenuation, which shows as photon starvation artifacts. Excluding the whole-body protocol and only considering standard FOV, helical fan beam kVCT (ClearRT) offers 33 different combinations of anatomical site, body size, and scan mode, which allows for a great range of customized and individualized imaging protocols. However, as the example in Figure 2 highlights, this also allows for inadequate imaging to be performed without standardized procedures, which will depend on treatment site, patient characteristics, and the purpose of the imaging study (localization/verification and/or adaptive planning).

All helical fan beam kVCT scans show windmill artifacts, which can readily be identified in the outer part of the body in Figure 1c and within the brain in Figure 3c. However, while noticeable, these artifacts do not reduce the usability of these images so long as the anatomy used for localization is not obscured by them. This similarly applies to the noisier and FOV limited kV-CBCT of the brain and head and neck regions (Fig. 2b and 3b). Furthermore, the craniotomy defect in the left posterior cranium is not visible on the helical fan beam kVCT, while both kV-CBCT and MVCT clearly identify it (Fig 3). Note that it did become visible, albeit smaller than on the other scans, when adjusting the window and level to only display bone and high CT numbers. This is likely due to the large slice thickness (as measured by the slice sensitivity profile) and the internal reconstruction algorithm that aims to enhance CNR at the expense of spatial resolution. Finally, none of the noncontrast enhanced setup imaging techniques are able to visualize the lesion in the left hemisphere that can clearly be seen on the contrast enhanced simulation CT scan. If these scans are to be used for plan adaptation one should choose acquisition parameters such that they allow for proper soft tissue matching and delineation and reduce artifacts inherent to the acquisition technique, as is the case with fine mode.

Except for spatial resolution and minimum achievable effective slice thickness, helical fan beam kVCT performs similarly to kV-CBCT at similar imaging dose levels. Similarly, all IQMs are considerably improved over those of MVCT,^14^ which together with the imaging dose reduction might potentially make MVCT obsolete for most cases, except those where imaging artifacts from high atomic number materials need to be avoided. In addition to improved IQMs, the use of helical fan beam kVCT is also expected to enable more accurate and precise adaptive monitoring and adaptive planning when registering the treatment helical kVCT to the simulation CT.^12^^,^^13^

## Conclusions

Helical fan beam kVCT enables the use of daily image guidance for localization and setup verification with comparable performance to existing kV-CBCT systems. With proper selection of scan parameters these scans may reduce the need for repeat CT simulation for replanning, reducing scheduling issues and workload and increasing patient experience. Improved image quality over MVCT also allows for increased precision of automated adaptive monitoring and planning tasks. Due to the large effective slice thicknesses for all parameter combinations these scans should not be used for simulation or planning of stereotactic procedures. Finally, imaging dose should be taken into consideration when designing standardized procedures for the use of helical fan beam kVCT, and selection of scan parameters should be guided by the desired use of each image set.

## Appendix Supplementary materials

Image, application 1
