# Supplementary material for: Clinical Assessment of a Novel Ring Gantry Linear Accelerator-Mounted Helical Fan-Beam kVCT System
Source: Adv Radiat Oncol. 2021 Dec 1;7(2):100862. doi: 10.1016/j.adro.2021.100862 (PMC8749200; doi:10.1016/j.adro.2021.100862)
Supplement: Supplementary file 1 [file mmc1.docx]

| **Table S1.** Basic ClearRT (fine mode) and CT simulator parameters for matched scans. Tube current (mA) is per view. Number of views depends on ClearRT scan mode. Imaging dose is reported as CTDIvol for 16 cm head (head) and 32 cm body (thorax and pelvis). For ClearRT normal and coarse mode multiply CTDIvol by 0.8 and 0.6, respectively. t_Slice_ and ∆_Slice_ are slice thickness and spacing, respectively. | | | | | | | | | | |
| --- | --- | --- | --- | --- | --- | --- | --- | --- | --- | --- |
| **Anatomical Size** | **Body Size** | **Voltage (kV)** | **Tube Current (mA)** | **t_Slice_ (mm)** | | **∆_Slice_ (mm)** | | **Pitch** | **CTDIvol (mGy)** | |
|  |  |  |  | **ClearRT** | **CT-Sim** | **ClearRT** | **CT Sim** |  | **ClearRT** | **CT Sim** |
| Head | Small | 100 | 80 | 2.40 | 2.50 | 1.20 | 2.50 | 0.75:1 | 11.0 | 12.3 |
|  | Medium | 100 | 125 |  |  |  |  |  | 17.2 | 19.1 |
|  | Large | 100 | 160 |  |  |  |  |  | 22.0 | 24.5 |
| Thorax | Small | 120 | 80 | 3.60 | 3.75 | 1.80 | 3.75 | 0.75:1 | 8.0 | 10.2 |
|  | Medium | 120 | 125 |  |  |  |  |  | 12.5 | 15.9 |
|  | Large | 120 | 160 |  |  |  |  |  | 16.0 | 20.4 |
|  | X-Large | 120 | 200 |  |  |  |  |  | 20.0 | 25.4 |
| Pelvis | Small | 140 | 80 | 3.60 | 3.75 | 1.80 | 3.75 | 0.75:1 | 11.5 | 14.0 |
|  | Medium | 140 | 125 |  |  |  |  |  | 18.0 | 21.9 |
|  | Large | 140 | 160 |  |  |  |  |  | 23.0 | 28.0 |
|  | X-Large | 140 | 200 |  |  |  |  |  | 28.8 | 37.1 |

| **Table S2.** CT simulator settings for clinically used simulation protocols for conventional and stereotactic body radiation therapy. | | | | | | |  |
| --- | --- | --- | --- | --- | --- | --- | --- |
| **Anatomical Site** | **Type** | **Tube Current**  **(mA)** | **Voltage**  **(kVp)** | **t_Slice_**  **(mm)** | **∆_Slice_**  **(mm)** | **Pitch** | **CTDI_vol_**  **(mGy)** |
| Brain | Helical | 280 | 120 | 2.5 | 2.5 | 1.5:1 | 25.3 |
| H&N | Helical | 300 | 120 |  |  |  | 27.2 |
| Thorax | Helical | 80 | 120 |  |  |  | 29.7 |
| Abdomen | Helical | 80 | 120 |  |  |  | 21.6 |
| Pelvis | Helical | 80 | 120 |  |  |  | 23.7 |
| SBRT | Axial | 420 | 120 | 1.25 | 1.25 | n/a | 67.0 |

| **Table S3.** kV-CBCT mode settings used clinically for setup imaging. | | | | | | |
| --- | --- | --- | --- | --- | --- | --- |
| **Machine** | **Site/Mode** | **Voltage**  **(kVp)** | **Tube Current**  **(mA)** | **Exposure**  **(mAs)** | **t_Slice_**  **(mm)** | **CTDI_vol_**  **(mGy)** |
|  |  |  |  |  |  |  |
| Halcyon | Head | 100 | 30 | 138 | 2 | 3.7 |
|  | Thorax | 125 | 35 | 301 | 2 | 6.0 |
|  | Pelvis | 125 | 80 | 1072 | 2 | 21.5 |
|  | Pelvis Large | 140 | 90 | 1454 | 2 | 38.5 |
| TrueBeam | Head | 100 | 15 | 150 | 2 | 3.2 |
|  | Thorax | 125 | 15 | 270 | 2 | 4.0 |
|  | Pelvis | 125 | 60 | 750 | 2 | 16.0 |
|  | Pelvis Large | 125 | 60 | 1074 | 2 | 37.0 |

| **Table S4.** Hounsfield units measured using the "Virtual Water" density phantom. A variance weighted average was computed from all three measurements. | | | | | | | | |
| --- | --- | --- | --- | --- | --- | --- | --- | --- |
| **Density Insert Name** | **Stated Density (g/cm^3^)** | **ClearRT** | | **Halcyon** | | **TrueBeam** | | **Weighted Average** |
|  |  | **Pelvis Large** | | **Pelvis** | | **Pelvis** | |  |
|  |  | **Mean** | $\boldsymbol{\sigma}$ | **Mean** | $\boldsymbol{\sigma}$ | **Mean** | $\boldsymbol{\sigma}$ |  |
| Cortical Bone (30%) | 1.33 | 498 | 10 | 370 | 20 | 551 | 22 | 484 |
| Cortical Bone (50%) | 1.56 | 870 | 11 | 721 | 23 | 904 | 24 | 852 |
| Cortical Bone (100%) | 1.82 | 1299 | 17 | 1172 | 34 | 1469 | 44 | 1294 |
| Inner Bone | 1.14 | 241 | 10 | 220 | 22 | 275 | 22 | 243 |
| LN 300 Lung | 0.28 | -697 | 15 | -701 | 25 | -751 | 29 | -707 |
| LN 450 Lung | 0.46 | -523 | 11 | -526 | 16 | -546 | 22 | -527 |
| Virtual Water (Inner) | 1.00 | 29 | 10 | -19 | 17 | 26 | 19 | 18 |
| Virtual Water (Outer) | 1.00 | 36 | 9 | -11 | 17 | 60 | 19 | 31 |
| True Water (Inner) | 1.00 | 19 | 10 | 28 | 20 | 75 | 21 | 30 |
| True Water (Outer) | 1.00 | 11 | 10 | -68 | 14 | 8 | 16 | -11 |
